# A conversation with Demilade Adedinsewo, M.B., Ch.B., Assistant Professor of Medicine, Mayo Clinic

**DOI:** 10.1017/cts.2024.639

**Published:** 2024-11-06

**Authors:** Clinical Research Forum

**Affiliations:** Clinical Research Forum, Washington, DC, USA

## Top 10 Clinical Research Achievement Awards Q & A

This article is part of a series of interviews with recipients of the Clinical Research Forum’s Top 10 Clinical Research Achievement Awards. This interview is with Demilade Adedinsewo, M.B., Ch.B., Assistant Professor of Medicine, Mayo Clinic. Dr Adedinsewo is a general noninvasive cardiologist with special interests in women’s heart health, cardiovascular disease prevention, cardiovascular health disparities, and the use of digital tools in cardiovascular disease management. She received a 2024 Top 10 Clinical Research Achievement Award for “Non-invasive detection of cardiac allograft rejection among heart transplant recipients using an electrocardiogram based deep learning model.” *The interview has been edited for length and clarity.*

## When did you first become interested in clinical research?

My interest in research began early in medical school. I was mostly influenced by my academic mentor at the time, Dr Adesegun Fatusi, who is a professor of public health with research experience working with the United Nations sexual and reproductive health agency (UNFPA). This was essentially my introduction to research methodology and how research findings can have a broad impact on population health. By the time I was done with medical school, I had decided on getting a master’s degree in public health (MPH). My MPH was in epidemiology and global health, and that experience also informed this path for me, helping me understand how I could be a clinician, but still do research and do it well.

## Your award-winning research involves artificial intelligence (AI). How did your research interests lead you to digital tools?

My work with digital tools started toward the end of my clinical cardiology fellowship, which was following graduate school and internal medicine residency training. I had the opportunity to work with a team evaluating AI and the electrocardiogram (ECG) for cardiac screening, and I started wondering if anyone was looking at this in unique patient populations such as pregnant and postpartum women. Working with my mentor, Dr Mohamad Yamani (senior author on the published manuscript), we discussed the possibility of looking at its use as a screening tool for heart transplant recipients.

## What were the findings of your award-winning research?

We found an AI-ECG model to be effective for detection of moderate-to-severe acute cellular rejection in heart transplant recipients.

## How can heart transplant patients benefit from this finding?

Our findings could improve transplant care by providing a rapid, noninvasive, and potentially remote screening option for cardiac allograft function. Right now, many heart transplant recipients still need to have frequent endomyocardial biopsies (EMB), particularly within the first year of transplant to screen for graft rejection. The procedure is inconvenient because it requires a trip to the hospital each time and is invasive, which means it’s not without risk. Those risks can range from mild, such as pericardial effusion, to severe, such as damage to a heart valve, which could require repeat cardiac surgery. The transplant community is very much interested in noninvasive options to screen for rejection among transplant recipients as this can improve care quality and decrease the burden of frequent invasive testing for patients. Two blood-based tests have been developed, but their effectiveness for detecting acute cellular rejection is somewhat limited. That’s why a tool like this could be so beneficial.

## What are the advantages of a remote screening option?

Some heart transplant patients are getting several biopsies (up to 14) within the first year. Due to the need for frequent testing, many patients often have to relocate for several months to be near a transplant facility. Contrast that with an ECG, which can be performed anywhere and then transmitted for review. Using an AI-ECG tool for testing could reduce the number of invasive procedures (and associated costs) and still let transplant cardiologists keep a close eye on their patients.

## How did you develop the AI-ECG model?

We wanted to know if AI could be trained to see subtle changes on an ECG that reflect acute cellular rejection after a heart transplant. We know that when a patient has severe rejection, there are marked changes on the ECG, but our hope was that we could use AI to identify subtle changes earlier. First, we did a retrospective study using data from heart transplant recipients with paired ECG and EMB results. We used this existing data to train and validate a working AI model for detection of allograft rejection. Then, we conducted a pilot prospective study, where we enrolled heart transplant recipients who had been scheduled for biopsies as part of their clinical care. Consenting patients had ECGs recorded, and AI prediction for rejection was generated; we then waited a few days for the biopsy results to be read so we could compare the results. The pathologists and members of the research study team were not privy to the AI-prediction results prior to the biopsy results. The AI test did an excellent job in correctly identifying all cases of rejection in the prospective study, although this was a small sample of 56 patients with only 2 cases of rejection. We are now comparing AI test results to the existing blood test methods, and the early results are showing it may perform better than the blood test. That’s very encouraging about the potential utility of this tool and whether in the future we should be combining it with a blood test to improve performance and ensure that we’re not missing any cases of rejection.

## What is the next step for this project?

Our work was limited to a Mayo Clinic patient population, and we’re learning from experience with other tools that sometimes, when these models are tested in different environments and with different patient populations, there could be differences in performance. So our next step is validating this model using existing data from other healthcare institutions that have matching ECG and biopsy data for heart transplant recipients. Subsequently, we will need to conduct a prospective study including a larger and diverse patient population.

## What else are you working on? Where is your research headed?

My key research focus is centered around developing and evaluating AI-powered technologies for cardiovascular screening with the goal of improving care for all patients. I especially want to address cardiovascular care disparities among women, particularly during pregnancy and postpartum. Heart disease, including heart failure, is a leading cause of death in the perinatal period, and early identification of heart failure in this population can be a huge diagnostic challenge. This is because many of the symptoms that we see with pregnancy often overlap with heart failure symptoms. As a result, the diagnosis of heart failure in pregnant and postpartum women is often delayed or missed, leading to adverse outcomes. An AI-ECG screening tool could be ideal for this population, and we’ve been able to demonstrate this in a retrospective study and a small pilot prospective study that it works in obstetric patients.

## What role does team science play in your research?

Team science plays a huge role. If we did not have the right team, I don’t believe this award-winning project would have happened. Our team included general cardiologists, cardiac electrophysiologists, transplant cardiologists, advanced heart failure experts, pathologists, biostatisticians, data scientists, clinical trainees, and research fellows. We are also incredibly grateful to the patients, relatives of patients, and hospital staff who participated in and or supported our research studies. The vast majority of enrolled participants (patients) were eager to participate and contribute data to the study and were also altruistic knowing that the results of the study would contribute to research knowledge and may not necessarily improve the care they were currently receiving. We got comments like: “I am happy to be a part of this, if it would help improve care for another patient!”

## What words of advice do you have for someone just beginning their career in clinical research?

As I see it, the key thing is being open to opportunities, especially when you’re starting out. Medicine tends to almost force you into a box. You pick a specialty, and then maybe a subspecialty, and there’s a tendency to be pigeonholed in your career as a clinician. You become an expert in something specific – which is great. But I find it’s also great to be open to different opportunities. My research trajectory wasn’t always in cardiovascular clinical research. I started in reproductive health and maternal health, and then with my epidemiology training, I ventured into infectious disease research, specifically HIV at the Centers for Disease Control and Prevention and then chronic disease prevention which included violence prevention in children, then nephrology, and finally ended up in the field of cardiovascular medicine. Each of those experiences helped me build a different skill and also gave me multidisciplinary experience. It all added up to what I’m doing today, and I learned so much just by being open to opportunities that came my way.

